# Risk factors of adult isoniazid-resistant and rifampicin-susceptible tuberculosis in Nanjing, 2019–2021

**DOI:** 10.1186/s12879-024-09404-y

**Published:** 2024-05-22

**Authors:** Jing Guo, Yan Han, Xia Zhang, Feishen Lin, Liangyu Chen, Xuebing Feng

**Affiliations:** 1 Department of Rheumatology and Immunology, Nanjing Drum Tower Hospital Clinical College of Nanjing University of Chinese Medicine, 321 Zhongshan Road, Nanjing, Jiangsu Province 210008 PR China; 2 Department of Tuberculosis, The Second Hospital of Nanjing, Nanjing University of Chinese Medicine, Nanjing, 211132 China

**Keywords:** Tuberculosis, Diabetes Mellitus, Hr-TB

## Abstract

**Introduction:**

This study aimed to analyze the risk factors associated with isoniazid-resistant and rifampicin-susceptible tuberculosis (Hr-TB) in adults.

**Method:**

The clinical data of 1,844 adult inpatients diagnosed with culture-positive pulmonary tuberculosis (PTB) in Nanjing Second Hospital from January 2019 and December 2021 were collected. All culture positive strain from the patient specimens underwent drug susceptibility testing (DST). Among them, 166 patients with Hr-TB were categorized as the Hr-TB group, while the remaining 1,678 patients were classified as having drug-susceptible tuberculosis (DS-TB). Hierarchical logistic regression was employed for multivariate analysis to identify variables associated with Hr-TB. Results: Multivariate logistic regression analysis revealed that individuals with diabetes mellitus (DM) (OR 1.472, 95% CI 1.037–2.088, *p* = 0.030) and a history of previous tuberculosis treatment (OR 2.913, 95% CI 1.971–4.306, *p* = 0.000) were at higher risk of developing adult Hr-TB, with this risk being more pronounced in male patients. Within the cohort, 1,640 patients were newly treated, and among them, DM (OR 1.662, 95% CI 1.123–2.461, *p* = 0.011) was identified as risk factors for Hr-TB. Conclusions: Diabetes mellitus is a risk factor for Hr-TB in adults, and the contribution of diabetes as a risk factor was more pronounced in the newly treatment or male subgroup. And previous TB treatment history is also a risk factor for Hr-TB in adults.

## Introduction

Based on Global Tuberculosis Report 2023, estimates of tuberculosis (TB) incidence rate in 2022 for China was 52 per 100,000 population [1]. These figures underscore China’s substantial TB burden. Isoniazid is a potent first-line anti-TB medication, and resistance to it increases the risk of treatment failure and relapse [2]. In 2018, the World Health Organization (WHO) released updated guidelines for the treatment of patients with isoniazid-resistant, rifampicin-susceptible TB (Hr-TB) [2]. Data reported to WHO from 156 countries or territories between 2002 and 2018 revealed that a relatively high proportion of TB patients had Hr-TB: 7.4% (95% CI 6.5–8.4%) of new patients and 11.4% (95% CI 9.4–13.4%) of previously treated patients [3]. Similarly, a recent systematic review of published literature indicated that the treatment of Hr-TB with the standard first-line regimen for new TB patients resulted in a treatment failure rate of 11% (95% CI 6–17%), compared to 2% (95% CI 1–3%) among drug-susceptible TB patients [4]. Adverse outcomes, such as treatment failure, death from any cause, loss to follow-up, and others, were significantly more frequent in patients with Hr-TB compared to those with drug-susceptible TB (DS-TB) [3]. The problem of unfavorable outcomes is particularly acute in eastern China, especially among previously treated patients [5]. Therefore, early diagnosis of Hr-TB plays a critical role in improving patient prognosis. To identify potential factors associated with Hr-TB, we collected and analyzed TB inpatient data from January 2019 to December 2021 at the Second Hospital of Nanjing.

## Methods

### Study area and study design

This retrospective study focused on the evaluation of inpatients with pulmonary tuberculosis (PTB) in the Nanjing district of China during the period from January 2019 to December 2021. As an infectious disease hospital in Nanjing, the Second Hospital of Nanjing is the only municipal designated medical institution for the diagnosis and treatment of tuberculosis and drug-resistant tuberculosis in Nanjing.

**Fig. 1:**
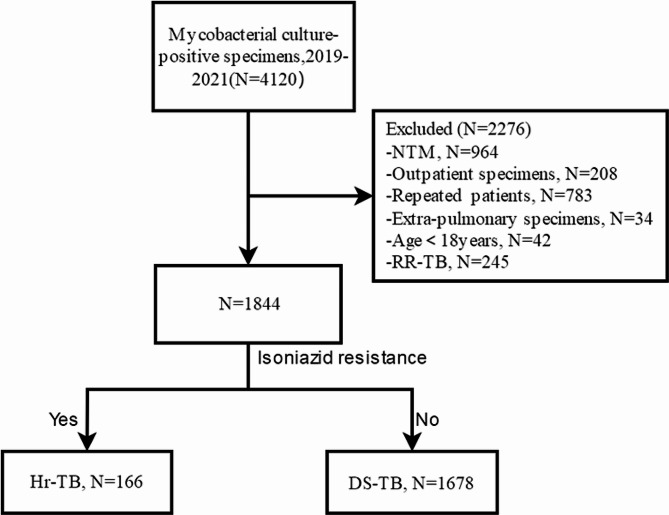
Flowchart of the study population Abbreviations: PTB, pulmonary tuberculosis; TB, tuberculosis; DS-TB, drug-susceptible tuberculosis; Hr-TB, Isoniazid-resistant tuberculosis; RR-TB, rifampicin-resistant tuberculosis

Initially, a total of 4,120 clinical specimens tested positive for mycobacterial culture and drug susceptibility testing (DST). Subsequently, several exclusions were made, including non-tuberculous mycobacteria strain (NTM) (964 strains), outpatient specimens (208 strains), repeatedly tested strains from the same patient (783 strains), strains from extra-pulmonary tissue specimens (34 strains), strains from patients under the age of 18 (42 strains), and rifampicin-resistant tuberculosis (RR-TB) cases strains (245 strains). Finally, 1,844 clinical isolates were included, including samples from sputum and bronchoalveolar lavage. (Fig. 1)

DS-TB is defined as an individual susceptible to both isoniazid and rifampin, which sets it apart from Hr-TB, where the latter is specifically resistant to isoniazid. Therefore, we identified 166 patients with Hr-TB who formed the Hr-TB group, while 1,678 patients with drug-susceptible tuberculosis (DS-TB) constituted the DS-TB group. (Fig. 1)

Trained research clinicians gathered demographic and clinical data, which encompassed information such as gender, age, smoking and drinking habits, race, body mass index (BMI), comorbidities (diabetes mellitus, HIV status, hypertension), history of previously tuberculosis treatment, site of tuberculosis infection (pulmonary or extrapulmonary), imaging characteristics (unilateral or bilateral tuberculosis, presence of lung cavities), serum albumin (ALB) levels, monocyte count, neutrophil count, and lymphocyte count. The following calculations were performed:

Prognostic Nutritional Index (PNI) was calculated as PNI = serum albumin (ALB) (g/L) + 5 × total lymphocyte count (10^9/L).

Neutrophil-to-lymphocyte ratio (NLR) was determined as the ratio of neutrophils to lymphocytes.

Monocyte-to-lymphocyte ratio (MLR) was calculated as the ratio of monocytes to lymphocytes.

Inclusion Criteria:

1. Adult inpatients diagnosed with pulmonary tuberculosis (PTB) [6].
2. Positive mycobacterial culture.
3. Availability of drug susceptibility test (DST) results.
4. Age ≥ 18 years patients.

Exclusion Criteria:

1. Non-tuberculous mycobacteria patient.
2. Extra-pulmonary tuberculosis patient without PTB.
3. Outpatients with incomplete clinical data.
4. Age < 18 years patient.
5. Rifampicin-resistant tuberculosis (RR-TB) patients.

### Laboratory methods

Mycobacteria were cultured using both liquid and solid media. The identification of mycobacterial strains followed the guidelines for clinical laboratory examination of tuberculosis in China. Drug susceptibility testing (DST) for anti-tuberculosis drugs was conducted on Middlebrook 7H10 agar plates using the absolute concentration method [7]. All culture media were procured from Zhuhai Beisuo Biotech Company.

### Quality control

Quality control measures were implemented in accordance with the guidelines outlined in the Rules for Laboratory Examination of Tuberculosis [7]. All procedures conducted during TB surveillance adhered to the guidelines provided by the World Health Organization (WHO) [1]. Quality assessment and data extraction were performed by a minimum of two researchers who had received professional training.

### Statistical analysis

Statistical analysis was carried out using SPSS version 26.0 for Microsoft Windows (SPSS Inc., http://www.spss.com.hk). A univariate logistic regression model was employed to identify factors associated with Hr-TB in PTB patients. In our binary logistic regression, the dependent variable was binary, with “1” indicating Hr-TB and “0” indicating DS-TB. For the analysis in the model, different demographic and clinical variables, namely gender, age, BMI, a history of DM, HIV status, a history of hypertension, TB retreatment, combined with extra-pulmonary tuberculosis, smoking history, alcohol consumption, Race, bilateral pulmonary infiltration, lung cavity, PNI, MLR, and NLR, have been treated as independent variables. Smoking history non-smoker and smoker (includes ex-smoker and current smoker). Alcohol consumption was divided into drinkers and non-drinkers. The continuous variables (age, BMI) were divided into groups (e.g. age: 18 ≤ age<60, ≥ 60;etc. BMI:<18.5, 18.5 ≤ BMI < 25, ≥ 25) follow standard WHO guideline [8]. The continuous variables (PNI, MLR) were divided into groups (e.g. PNI: PNI<40, ≥ 40; etc. MLR: <0.5, ≥ 0.5).

Before conducting binary logistic regression analysis, perform univariate logistic regression to preliminarily screen for predictive risk factors for Hr-TB, eliminating potentially insignificant variables with a significance threshold of *p* < 0.1. Subsequently, construct a multiple-factor Logistic model using a stepwise forward selection approach.

There were some confounding factors. In order to explore the impact of diabetes as a single factor on outcomes, we will conduct subgroup analyses according to gender and treatment history to control the method of dealing with confounding.

## Results

In total, 1844 strains DST positive for tuberculosis were interviewed for the survey. It divided in two groups: Hr-TB and DS-TB. A total of 166 patients were included in the Hr-TB group, including 125 newly cases and 41 previously treatment cases, and 121 cases of male (121/166, 72.9%). There were 1678 cases in the DS-TB group (including 1515 newly cases and 163 previously treatment cases), and 1180 cases (1180/1678, 70.3%) of males. There were no significant differences in age, gender, BMI, smoking, alcohol, ethnicity, and other relevant demographic factors between the DS-TB group and the Hr-TB group. There was a significant difference in the proportion of patients with pulmonary tuberculosis who had previously TB treatment between the two groups (*p* = 0.000) (refer to Table 1).

**Table 1:** Univariate logistic regression analysis of risk factor for Hr-TB in PTB patients

Variable	Hr-TB (*n* = 166)	DS-TB (*n* = 1678)	Unadjusted OR (95% CI)	*P* value
Male, n (%)	121 (72.9%)	1180 (70.3%)	1.135 (0.793–1.623)	0.489
Age, Median (IQR)	55 (38.00–64.00)	51 (30.00–65.00)	1.007 (0.999–1.015)	0.105
Age group, n (%)				
18–59	101 (60.8%)	1085 (64.7%)		
>=60	65 (39.2%)	593 (35.3%)	1.178 (0.849–1.634)	0.328
BMI (kg/m2), Median (IQR)	20.35 (18.79–22.77)	20.20 (18.37–22.47)	1.032 (0.981–1.085)	0.225
BMI group, n (%)				
< 18.5, n (%)	38 (22.9%)	447 (26.6%)	0.861 (0.586–1.266)	0.447
18.5 - <25, n (%)	108 (65.1%)	1094 (65.2%)		0.179
≥ 25, n (%)	20 (12.0%)	137 (8.2%)	1.479 (0.889–2.460)	0.132
DM, n (%)	58 (34.9%)	420 (25.0%)	1.609 (1.148–2.255)	0.006*
HIV status, n (%)	0 (0.0%)	15 (0.9%)	0.000 (0.000-)	0.999
Hypertension, n (%)	28 (16.9%)	277 (16.5%)	1.026 (0.670–1.572)	0.905
Tuberculosis treatment, n (%)	41 (24.7%)	163 (9.7%)	3.049 (2.068–4.494)	0.000*
PTB and EPTB, n (%)	13 (7.8%)	199 (11.9%)	0.631 (0.352–1.134)	0.124
Smoking, n (%)	44 (26.5%)	441 (26.3%)	1.012 (0.705–1.452)	0.950
Alcohol, n (%)	28 (16.9%)	232 (13.8%)	1.265 (0.823–1.943)	0.284
Race (Han), n (%)	163 (98.2%)	1654 (98.6%)	0.788 (0.235–2.646)	0.700
Bilateral pulmonary infiltration, n (%)	129 (77.7%)	1272 (75.8%)	1.113 (0.759–1.631)	0.584
Lung Cavity, n (%)	86 (51.8%)	739 (44.0%)	1.366 (0.993–1.880)	0.056
PNI, Median (IQR)	44.48 (38.93–48.70)	43.90 (38.20-48.75)	1.000 (0.992–1.007)	0.913
PNI ≥ 40, n (%)	118 (71.1%)	1142 (68.1%)	1.154 (0.812–1.639)	0.424
MLR, Median (IQR)	0.43 (0.29–0.63)	0.44 (0.30–0.67)	0.798 (0.504–1.264)	0.337
MLR ≥ 0.5, n (%)	68 (41.0%)	731 (43.6%)	0.899 (0.650–1.243)	0.519
NLR, Median (IQR)	3.57 (2.41–5.19)	3.49 (2.32–5.84)	0.972 (0.936–1.010)	0.147

Abbreviations: BMI, body mass index; OR, odds ratio; EPTB, extra-pulmonary tuberculosis; PNI, prognostic nutritional index= [serum albumin (ALB) (g/L) + 5 × total lymphocyte count (109/L)]; NLR, neutrophil-to-lymphocyte ratio; MLR, monocyte-to-lymphocyte ratio; FBG, fasting blood glucose; Data are median (IQR), n (%), or n/N (%). *p < 0.05

### Univariate logistics regression analysis of risk factors for Hr-TB

Through univariate logistic regression analysis, it was determined that gender, age, age group, BMI, BMI group, HIV status, PTB combined with extra-pulmonary tuberculosis, smoking, alcohol consumption, ethnicity, PNI, PNI ≥ 40, MLR, MLR ≥ 0.5, and NLR were not significantly associated with Hr-TB. However, diabetes mellitus and a history of previous tuberculosis treatment showed a strong association with Hr-TB, with odds ratios of (OR 1.609, 95% CI 1.148–2.255, *p* = 0.006) and (OR 3.049, 95% CI 2.068–4.494, *p* = 0.000), respectively. Additionally, there was weak evidence suggesting that the presence of a lung cavity might be associated with an increased risk of acquiring Hr-TB (OR 1.366, 95% CI 0.993–1.880, *p* = 0.056), although this result just fell short of reaching statistical significance (refer to Table 1).

### Multivariate logistic regression analyzes risk factors for Hr-TB

Following the univariate logistic analysis, we incorporated diabetes, the presence of a lung cavity, and a history of previous tuberculosis treatment into the binary regression equation, which was established using the enter method. The results demonstrated that diabetes (OR 1.472, 95% CI 1.037–2.088, *p* = 0.030) and a history of previous tuberculosis treatment (OR 2.913, 95% CI 1.971–4.306, *p* = 0.000) are significant risk factors for adult Hr-TB in Nanjing (refer to Table 2).

**Table 2:** Multivariate logistic regression analysis of risk factor for Hr-TB in PTB patients

Variable	B	S.E.	Wald	Adjusted OR (95% CI)	*P* value
diabetes(1)	0.386	0.178	4.689	1.472 (1.037–2.088)	0.030*
Tuberculosis treatment(1)	1.069	0.199	28.746	2.913 (1.971–4.306)	0.000*
Lung Cavity(1)	0.176	0.169	1.082	1.192 (0.856–1.660)	0.298

### Analysis of risk factor for Hr-TB in different subgroup

In the subgroup analysis of male patients, a multivariate analysis revealed compelling evidence that both diabetes (OR 1.555, 95% CI 1.055–2.291, *p* = 0.026) and a history of previous tuberculosis treatment (OR 3.120, 95% CI 2.022–4.814, *p* = 0.000) were significantly associated with an elevated risk of Hr-TB infection (see Table 3). In the subgroup analysis of female patients, BMI ≥ 25 also exhibit an increased risk of Hr-TB.

**Table 3:** Univariate and multivariate logistic regression analysis of risk factor for Hr-TB in different stratification

Variable	Hr-TB (*n* = 166)	DS-TB (*n* = 1678)	Unadjusted OR (95% CI)	*P* value	Adjusted OR (95% CI)	*P* value
**Male, n (%)**	121 (72.9%)	1180 (70.3%)				
Age, Median (IQR)	56.00 (44.00-65.50)	55.00 (34.00–67.00)	1.005 (0.995–1.016)	0.295		
Age group, n (%)						
18–59	69 (57.0%)	701 (59.4%)				
>=60	52 (43.0%)	479 (40.6%)	1.103 (0.756–1.610)	0.612		
BMI (kg/m2), Median (IQR)	20.76 (18.98–22.77)	20.40 (18.50-22.68)	1.026 (0.967–1.087)	0.398		
BMI group, n (%)						
< 18.5, n (%)	25 (20.7%)	296 (25.1%)	0.789 (0.494–1.258)	0.319		
18.5 - <25, n (%)	83 (68.6%)	775 (65.7%)		0.529		
≥ 25, n (%)	13 (10.7%)	109 (9.2%)	1.114 (0.600-2.066)	0.733		
DM, n (%)	50 (41.3%)	360 (30.5%)	1.604 (1.094–2.351)	0.015*	1.555 (1.055–2.291)	0.026*
HIV status, n (%)	0 (0.0%)	15 (1.3%)	0.000 (0.000-)	0.999		
Hypertension, n (%)	20 (16.5%)	221 (18.7%)	0.859 (0.520–1.419)	0.553		
Tuberculosis treatment, n (%)	35 (28.9%)	134 (11.4%)	3.177 (2.062–4.894)	0.000*	3.120 (2.022–4.814)	0.000*
PTB and EPTB, n (%)	9 (7.4%)	139 (11.8%)	0.602 (0.298–1.214)	0.156		
Smoking, n (%)	43 (35.5%)	370 (31.4%)	1.207 (0.815–1.786)	0.347		
Alcohol, n (%)	26 (21.5%)	188 (15.9%)	1.444 (0.911–2.290)	0.118		
Race (Han), n (%)	119 (98.3%)	1169 (99.1%)	0.560 (0.123–2.556)	0.454		
Bilateral pulmonary infiltration, n (%)	98 (81.0%)	927 (78.6%)	1.163 (0.723–1.870)	0.533		
Lung Cavity, n (%)	66 (54.5%)	584 (49.5%)	1.225 (0.841–1.783)	0.290		
PNI, Median (IQR)	42.95 (37.38-48.00)	44.45 (38.90-48.68)	1.000 (0.993–1.007)	0.966		
PNI ≥ 40, n (%)	84 (69.4%)	749 (63.5%)	1.306 (0.872–1.958)	0.195		
MLR, Median (IQR)	0.49 (0.31–0.70)	0.50 (0.33–0.72)	0.844 (0.513–1.388)	0.504		
MLR ≥ 0.5, n (%)	60 (49.6%)	602 (51.0%)	0.944 (0.650–1.373)	0.764		
NLR, Median (IQR)	4.00 (2.51–5.77)	3.88 (2.56–6.47)	0.970 (0.929–1.012)	0.970		
**Female, n (%)**	45 (27.1%)	498 (29.7%)				
Age, Median (IQR)	46.00 (27.50–61.00)	35.00 (26.00–58.00)	1.009 (0.994–1.025)	0.243		
Age group, n (%)						
18–59	32 (71.1%)	384 (77.1%)				
>=60	13 (28.9%)	114 (22.9%)	1.368 (0.695–2.695)	0.364		
BMI (kg/m2), Median (IQR)	19.92 (17.73–22.95)	19.81 (18.20-21.73)	1.046 (0.946–1.157)	0.381		
BMI group, n (%)						0.043*
< 18.5, n (%)	13 (28.9%)	151 (30.3%)	1.099 (0.547–2.207)	0.792	1.154 (0.569–2.343)	0.691
18.5 - <25, n (%)	25 (55.6%)	319 (64.1%)		0.045		
≥ 25, n (%)	7 (15.6%)	28 (5.6%)	3.190 (1.268–8.028)	0.014	3.340 (1.297–8.601)	0.012*
DM, n (%)	8 (17.8%)	60 (12.0%)	1.578 (0.702–3.550)	0.270		
HIV status, n (%)	-	-	-	-		
Hypertension, n (%)	8 (17.8%)	56 (11.2%)	1.707 (0.757–3.848)	0.198		
Tuberculosis treatment, n (%)	6 (13.3%)	29 (5.8%)	2.488 (0.974–6.355)	0.057	2.133 (0.812–5.606)	0.124
PTB and EPTB, n (%)	4 (8.9%)	60 (12%)	0.712 (0.246–2.059)	0.531		
Smoking, n (%)	1 (2.2%)	71 (14.3%)	0.137 (0.019–1.008)	0.051	0.149 (0.020–1.105)	0.063
Alcohol, n (%)	2 (4.4%)	44 (8.8%)	0.480 (0.112–2.048)	0.321		
Race (Han), n (%)	44 (97.8%)	485 (97.4%)	1.179(0.151–9.228)	0.875		
Bilateral pulmonary infiltration, n (%)	31 (68.9%)	345 (69.3%)	0.982 (0.508–1.898)	0.957		
Lung Cavity, n (%)	20 (44.4%)	155 (31.1%)	1.770 (0.954–3.284)	0.070	1.741 (0.920–3.293)	0.088
PNI, Median (IQR)	44.85 (40.10-49.45)	45.68 (41.10-49.41)	0.992 (0.949–1.038)	0.733		
PNI ≥ 40, n (%)	34 (75.6%)	393 (78.9%)	0.826 (0.405–1.685)	0.599		
MLR, Median (IQR)	0.36 (0.24–0.45)	0.35 (0.26–0.50)	0.373 (0.082–1.690)	0.201		
MLR ≥ 0.5, n (%)	8 (17.8%)	129 (25.9%)	0.618 (0.281–1.363)	0.233		
NLR, Median (IQR)	2.84 (1.94–4.04)	2.80 (1.99–4.18)	0.965 (0.867–1.075)	0.523		
**Newly treatment, n (%)**	125 (75.3%)	1515 (90.3%)				
Male, n (%)	86 (68.8%)	1046 (69.0%)	0.989 (0.667–1.466)	0.955		
Age, Median (IQR)	52.00 (34.50–63.50)	49.00 (29.00–64.00)	1.004 (0.995–1.013)	0.401		
Age group, n (%)						
18–59	78 (62.4%)	1005 (66.3%)				
>=60	47 (37.6%)	510 (33.7%)	1.187 (0.814–1.732)	0.372		
BMI (kg/m2), Median (IQR)	20.51 (18.84–22.77)	20.20 (18.38-22.46)	1.040 (0.981–1.102)	0.193		
BMI group, n (%)				0.123		0.517
< 18.5, n (%)	27 (21.6%)	393 (25.9%)	0.840 (0.536–1.318)	0.449	0.951 (0.599–1.508)	0.830
18.5 - <25, n (%)	82 (65.6%)	1003 (66.2%)				
≥ 25, n (%)	16 (12.8%)	119 (7.9%)	1.645 (0.932–2.903)	0.086	1.379 (0.771–2.470)	0.279
DM, n (%)	42 (33.6%)	369 (24.4%)	1.572 (1.065–2.319)	0.023*	1.582 (1.056–2.370)	0.026*
HIV status, n (%)	0 (0.0%)	15 (1.0%)	0.000 (0.000-)	0.999		
Hypertension, n (%)	19 (15.2%)	243 (16.0%)	0.938 (0.565–1.558)	0.806		
PTB and EPTB, n (%)	9 (7.2%)	178 (11.7%)	0.583 (0.291–1.169)	0.128		
Smoking, n (%)	30 (24%)	391 (25.8%)	0.908 (0.593–1.390)	0.656		
Alcohol, n (%)	19 (15.2%)	204 (13.5%)	1.152 (0.692–1.918)	0.587		
Race (Han), n (%)	122 (97.6%)	1493 (98.5%)	0.599 (0.177–2.030)	0.411		
Bilateral pulmonary infiltration, n (%)	94 (75.2%)	1128 (74.5%)	1.040 (0.682–1.587)	0.854		
Lung Cavity, n (%)	61 (48.8%)	652 (43.0%)	1.262 (0.876–1.818)	0.212		
PNI, Median (IQR)	44.70 (40.83–48.85)	44.20 (38.50–48.90)	1.000 (0.994–1.007)	0.937		
PNI ≥ 40, n (%)	96 (76.8%)	1052 (69.4%)	1.457 (0.948–2.238)	0.086	1.207 (0.738–1.974)	0.453
MLR, Median (IQR)	0.41 (0.28–0.58)	0.43 (0.30–0.66)	0.607 (0.333–1.105)	0.102		
MLR ≥ 0.5, n (%)	46 (36.8%)	647 (42.7%)	0.781 (0.536–1.139)	0.200		
NLR, Median (IQR)	3.32 (2.20–5.03)	3.40 (2.30–5.58)	0.946 (0.895-1.000)	0.052	0.955 (0.897–1.016)	0.146
**Previously treatment**	41 (24.7%)	163 (9.7%)				
Male, n (%)	35 (85.4%)	134 (82.2%)	1.262 (0.486–3.279)	0.632		
Age, Median (IQR)	58.00 (49.00-68.50)	60.00 (47.00–72.00)	0.998 (0.978–1.019)	0.885		
Age group, n (%)						
18–59	23 (56.1%)	80 (49.1%)				
>=60	18 (43.9%)	83 (50.9%)	0.754 (0.379–1.502)	0.423		
BMI (kg/m2), Median (IQR)	20.28 (18.01–22.25)	19.82 (17.72–22.49)	1.028 (0.933–1.132)	0.581		
BMI group, n (%)				0.837		
< 18.5, n (%)	11 (26.8%)	50 (30.7%)	0.804 (0.367–1.760)	0.585		
18.5 - <25, n (%)	26 (63.4%)	95 (58.3%)				
≥ 25, n (%)	4 (9.8%)	18 (11.0%)	0.812 (0.253–2.608)	0.726		
DM, n (%)	16 (39%)	51 (31.3%)	1.405 (0.691–2.857)	0.347		
HIV status, n (%)	-	-	-	-		
Hypertension, n (%)	9 (22.0%)	34 (20.9%)	1.067 (0.465–2.448)	0.878		
PTB and EPTB, n (%)	4 (9.8%)	21 (12.9%)	0.731 (0.236–2.260)	0.586		
Smoking, n (%)	14 (34.1%)	50 (30.7%)	1.172 (0.567–2.423)	0.669		
Alcohol, n (%)	9 (22.0%)	28 (17.2%)	1.356 (0.583–3.154)	0.479		
Race (Han), n (%)	41 (100%)	161 (98.8%)	411394631.1 (0.000-)	0.999		
Bilateral pulmonary infiltration, n (%)	35 (85.4%)	144 (88.3%)	0.770 (0.286–2.070)	0.604		
Lung Cavity, n (%)	25 (61.0%)	87 (53.4%)	1.365 (0.679–2.746)	0.383		
PNI, Median (IQR)	40.65 (35.53–47.85)	41.25 (35.20–45.80)	1.013 (0.969–1.059)	0.567		
PNI ≥ 40, n (%)	22 (53.7%)	90 (55.2%)	0.939 (0.472–1.867)	0.858		
MLR, Median (IQR)	0.53 (0.34–0.76)	0.52 (0.33–0.80)	0.955 (0.436–2.092)	0.908		
MLR ≥ 0.5, n (%)	22 (53.7%)	84 (51.5%)	1.089 (0.548–2.163)	0.808		
NLR, Median (IQR)	4.14 (2.94–7.48)	4.55 (2.89–7.35)	0.976 (0.922–1.034)	0.407		

Note: BMI classification criteria: underweight (BMI < 18.5 kg/m2), normal weight (BMI 18.5 to < 25 kg/m2), overweight or obese (BMI ≥ 25 kg/m2).

Furthermore, within the subgroup of newly diagnosed tuberculosis patients, a multivariate analysis indicated that diabetes (OR 1.582, 95% CI 1.056–2.370, *p* = 0.026) was associated with an increased risk of Hr-TB infection.

However, in the subgroup of patients with a history of previous tuberculosis treatment, there was insufficient evidence to suggest that diabetes was associated with an increased risk of Hr-TB infection (refer to Table 3).

## Discussion

Isoniazid is a critical first-line drug used in the treatment of tuberculosis (TB) and latent TB infection. Resistance to isoniazid can significantly impact the effectiveness of TB treatment. Globally, in 2019, 11% (with a range of 6.5–15%) of all incident TB cases were attributed to isoniazid-resistant TB (Hr-TB) [1]. Isoniazid resistance primarily stems from mutations in genes such as katG, the promoter region of inhA, and the promoter region of ahpC. Other mechanisms, including the up-regulation of efflux pumps and reduced drug concentrations, may also contribute to isoniazid resistance [9, 10]. Therefore, gaining early insights into the incidence of Hr-TB and its associated risk factors is crucial for enhancing the overall success rate of TB treatment.

The findings from this study highlight that a history of previous tuberculosis treatment and diabetes mellitus (DM) serve as risk factors for adult isoniazid-resistant tuberculosis (Hr-TB) in Nanjing. Patients with a prior history of TB treatment often exhibit poorer treatment outcomes, higher mortality rates, and an elevated likelihood of developing drug resistance [11]. Notably, multidrug-resistant TB (MDR-TB) occurs 5–10 times more frequently among individuals who have previously undergone TB treatment compared to those with new TB cases [1]. The substantial burden of drug resistance among previously treated TB patients in Uganda underscores the potential for the emergence of additional drug resistance [12]. These findings align with and support the outcomes of the present study.

Both type 1 and type 2 diabetes mellitus (DM) have been associated with an increased risk of active tuberculosis (TB) and multidrug-resistant TB (MDR-TB) [13, 14]. DM has been identified as a risk factor for various forms of drug resistance, including polydrug resistance (PDR), streptomycin (SM) resistance, and isoniazid (INH) combined with SM resistance, particularly among newly diagnosed TB cases [14]. Nevertheless, a Taiwanese study reported that DM did not elevate the risk of multidrug resistance, possibly due to inadequate adjustment for potential confounding factors such as HIV status, smoking habits, and alcohol consumption [15, 16]. These findings suggest that the development of drug resistance may be influenced by factors like comorbidities or the level of glycemic control in individuals with DM. Therefore, we conducted logistic regression analysis, incorporating common risk factors that affect drug-resistant TB and excluding potential interfering factors. The statistical analysis revealed that DM was indeed a risk factor for adult Hr-TB patients.

Interestingly, in the subgroup analysis of retreatment cases, diabetes was not identified as an independent risk factor for Hr-TB. In a retrospective study of previously treated pulmonary tuberculosis in Shandong, China, the highest proportion of comorbidity was found for DM (9.5%), followed by hypertension (2.0%) and COPD (1.8%), and comorbidity was significantly associated with overall INH (OR: 1.62, 95% CI 1.16 to 2.26) in univariable analysis (*p* < 0.05), but not in multivariable analysis (*p* > 0.05) [17]. The results of the two papers are consistent. Patients in the retreated TB group may be diabetic patients with known good glycemic control, and in newly diagnosed TB patients with poor glycemic control, they may be known diabetic patients. The reason for this observation needs to be further explored. It’s worth noting that diabetic individuals with factors such as a high bacterial load, reduced effective drug concentrations of anti-tuberculosis medications, compromised host immune responses, and decreased levels of IFN-γ and IL-12 can be more susceptible to Mycobacterium tuberculosis (MTB) infection and at a heightened risk of drug resistance [16]. Existing literature has corroborated the association between diabetes and genotypic resistance to at least one anti-tuberculosis drug, as determined by a Logit multivariate regression model [18]. Studies have also indicated that elevated glucose levels in TB patients with diabetes can lead to delayed absorption and faster elimination of isoniazid (INH), resulting in lower plasma concentrations of INH [19, 20]. Furthermore, the HbA1c level has been established as an independent risk factor for isoniazid resistance and multidrug resistance in TB patients with comorbid type 2 diabetes mellitus (T2DM) [21]. The observation that diabetes is not a risk factor for Hr-TB in retreated patients raises questions about potential associations with factors like blood glucose control levels and genetic predispositions, warranting further investigation.

Based on the previous theory, we conducted subgroup analyses by gender. In the whole data analysis and the male subgroup data analysis diabetes were statistically significant as a risk factor for Hr-TB, but there was no statistical significance in the female subgroup, and the reason for this phenomenon was that there was an interaction between gender and diabetes, and the proportion of diabetic patients in men was higher than that in women [22], adult males show higher rates of TB than females [23, 24], and men mainly showed impaired fasting blood glucose, while women usually showed impaired glucose tolerance [25]. Whether impaired fasting blood glucose is a risk factor for Hr-TB in diabetic patients needs to be further studied and explored.

This study has certain limitations. We did not have access to data on molecular resistance genes, diabetic glycemic control levels, immunotrophic markers, and other relevant indicators in patients with Hr-TB. And we don’t include some variables like course of treatment, COVID-19, SES, non-completion and failure of TB treatment, adverse drug reaction, non-adherent, and COPD as these are important factors as well. For example, the COVID-19 pandemic has been effective in controlling the transmission of TB, but it has been detrimental to the control of T2DM [26].

Consequently, we were unable to explore additional potential risk factors for Hr-TB or the reasons why diabetes was not identified as a risk factor in patients undergoing retreatment. In the future, expanding the sample size could be a valuable step to further investigate these aspects.

## Conclusion

This study found that Diabetes mellitus is a risk factor for Hr-TB in adults, and the contribution of diabetes as a risk factor was more pronounced in the newly treatment or male subgroup. And previous TB treatment history is also a risk factor for Hr-TB in adults. These findings enhance our understanding of Hr-TB in PTB-DM patients, facilitating early drug resistance screening and the development of rational treatment strategies. Future research should focus on analyzing the shared genetic resistance factors between DM and Hr-TB to further inform clinical management and interventions.

## Data Availability

The datasets generated and analyzed during the current study are not publicly available due to the fact that it contains personal information, but are available from the corresponding author on reasonable request.

## Abbreviations

TB: Tuberculosis
PTB: Pulmonary tuberculosis
MTB: Mycobacterium tuberculosis
DM: Diabetes mellitus
T2DM: Type 2 diabetes mellitus
Hr-TB: Isoniazid-resistant and rifampicin-susceptible TB
RR-TB: Rifampicin-resistant tuberculosis
MDR-TB: Multidrug-resistant tuberculosis
PTB-DM: Pulmonary tuberculosis with diabetes mellitus
DST: Drug susceptibility test
