# Correction: Risk factors of adult isoniazid-resistant and rifampicin-susceptible tuberculosis in Nanjing, 2019–2021

**DOI:** 10.1186/s12879-024-09632-2

**Published:** 2024-07-22

**Authors:** Jing Guo, Yan Han, Xia Zhang, Feishen Lin, Liangyu Chen, Xuebing Feng

**Affiliations:** 1 Department of Rheumatology and Immunology, Nanjing Drum Tower Hospital Clinical College of Nanjing University of Chinese Medicine, 321 Zhongshan Road, Nanjing, Jiangsu Province 210008 PR China; 2 Department of Tuberculosis, The Second Hospital of Nanjing, Nanjing University of Chinese Medicine, Nanjing, 211132 China

**Guo et al. BMC Infectious Diseases (2024) 24:511**

10.1186/s12879-024-09404-y

Following publication of the original article [1], we have been notified that the order of the author affiliations was incorrect.

Initially, Department of Tuberculosis at The Second Hospital of Nanjing was presented as affiliation number one, while Department of Rheumatology and Immunology at Nanjing Drum Tower Hospital was presented as affiliation number two. This has now been switched.

The original article has been corrected.
